# ﻿A new species of *Piper* (Piperaceae) with peltate leaves from Serranía de las Quinchas, Colombia

**DOI:** 10.3897/phytokeys.227.101405

**Published:** 2023-05-29

**Authors:** M. Alejandra Jaramillo, Dayro Rodríguez-Duque, Magda Escobar-Alba

**Affiliations:** 1 Grupo Diversitas, Facultad de Ciencias Básicas y Aplicadas, Universidad Militar Nueva Granada, km 2 Via Cajicá-Zipaquirá, Cajicá, Colombia Universidad Militar Nueva Granada Cajicá Colombia; 2 Grupo de Investigación Biodiversidad y Conservación, Museo de Historia Natural “Luis Gonzalo Andrade”, Facultad de Ciencias, Universidad Pedagógica y Tecnológica de Colombia-Universidad Nacional de Colombia, Av. Central del Norte 39-115, Tunja-Boyacá, Tunja, Colombia Universidad Pedagógica y Tecnológica de Colombia-Universidad Nacional de Colombia Tunja Colombia

**Keywords:** Boyacá, Chocó Region, *Macrostachys* clade, Piperales, tropical montane forests

## Abstract

*Piperquinchasense* is described and illustrated as a new species occurring in the understory of wet montane forest of the middle Magdalena Valley in Colombia, the easternmost portion of the Chocó Region. Its relationships are discussed with related taxa from the *Macrostachys* clade. An identification key for 35 Neotropical *Piper* species with peltate leaves is provided.

## Abstract (es)

*Piperquinchasense* se describe e ilustra como una nueva especie que ocurre en el sotobosque de bosques húmedos montanos del valle medio del Magdalena en Colombia, la porción más oriental del Chocó Biogeográfico. Se discuten sus relaciones con otras especies del clado *Macrostachys*. Se presenta una clave de identificación para 35 especies de *Piper* Neotropical con hojas peltadas.

## ﻿Introduction

*Piper*, with more than 2000 species (Callejas-Posada 2020), is one of the most species-rich genera among flowering plants (Frodin 2004). *Piper* is also a common element in the understory of Neotropical forests (Gentry 1990; Draper et al. 2021). Species of *Piper* are a key resource for bats of the genus *Carollia* (Fleming 2004; Bohlender et al. 2018; Yohe et al. 2021), and they are a critical element that supports diverse trophic networks that involve moths and parasitoid wasps (Wilson et al. 2012; Slinn et al. 2018). *Piper*’s enormous diversity of secondary metabolites is critical for their coexistence (Salazar et al. 2016a, 2016b) and diversification (Massad et al. 2022). And it is also of immense interest in the pharmaceutical industry (Perez Gutierrez et al. 2013; Salehi et al. 2019). This genus is, without doubt, an essential structural and trophic element of the understory and lower strata of Neotropical forests (Salazar et al. 2016b; Draper et al. 2021).

Molecular phylogenetic studies have been instrumental in reviving the infrageneric classification of the genus, but also identifying convergence in morphological traits (Jaramillo and Callejas-Posada 2004; Trujillo et al. 2022). Molecular phylogenetics (Jaramillo and Manos 2001; Jaramillo et al. 2008) have validated the monophyly of groupings proposed in the mid-1800s (Kunth 1839; Miquel 1843). The infrageneric classification was not used during the 1900s, probably as the expeditions in the early 1900s produced too many species to classify. Today we know that *Piper* clades and subclades are easy to recognize with a combination of key morphological characters (Jaramillo et al. 2008). A formal infrageneric classification of *Piper* based on phylogeny is under preparation (Callejas pers. comm.). Molecular phylogenetics have been instrumental in clarifying the relationships of *Piper* species with “axillary” inflorescences, a polyphyletic set of taxa from the Chocó Region that were wrongly merged in the genus *Trianaeopiper* Trel. (Trelease 1928). Molecular phylogenetics demonstrated that *Trianaeopiper* is not monophyletic, revealing that the diagnostic character axillary inflorescence – which are shortened sympodial branches, is convergent (Jaramillo and Callejas-Posada 2004). Similarly, molecular phylogenetics have further supported that *Piper* species with peltate leaves are part of at least four Neotropical *Piper* clades (Trujillo et al. 2022). *Piper* classification, evolution and ecological studies have been greatly enriched with molecular phylogenetics.

Identifying *Piper* species continues to be difficult for the untrained eye, and many taxa are submerged in a few large, broadly distributed, but artificial taxa (Tebbs 1990, 1993). To the trained specialist, new *Piper* species are a common finding in the tropical forests, or even in the undetermined piles in the most important herbaria around the world. Additionally, molecular phylogenetics have confirmed the clade (subgeneric) affiliation of new species (Bornstein et al. 2014; Tepe et al. 2014; Trujillo et al. 2022). During our exploration to Serranía de las Quinchas we have identified a few undescribed species, one of the stands-out because it has peltate leaves, a characteristic rarely observed in the genus.

“Serranía de las Quinchas” is a small mountain spur west of the Cordillera Oriental in the middle Magdalena Valley, in Colombia. The region’s flora is particularly interesting as it combines its own floristic elements, mixed with taxa from Mesoamerica, the Chocó Region, and Amazonia (Balcázar-Vargas et al. 2000). Additionally, various endemic species and genera occur in the area (Rodríguez-Duque et al. 2021). The middle Magdalena Valley deserves close examination and more fieldwork to uncover the history of its biological richness. For centuries, the forest has been preserved because of its scarp ridges and high rainfall. However, the construction of a pipeline and road in the 1990s opened the area to colonization (Stiles and Bohórquez 2000). Today the region is threatened by the extension of agriculture and mining activities. Fortunately, Serranía de las Quinchas Regional Park was created in 2008 by Corpoboyacá (the regional environmental protection agency) to protect the remaining forest (Bohorquez-Osorio et al. 2020). Despite the deforestation in the region, new species are still being encountered. Here we describe a new species of Neotropical *Piper* with peltate leaves that we detected during our expeditions in this interesting location.

## ﻿Materials and methods

**Figure 1.**
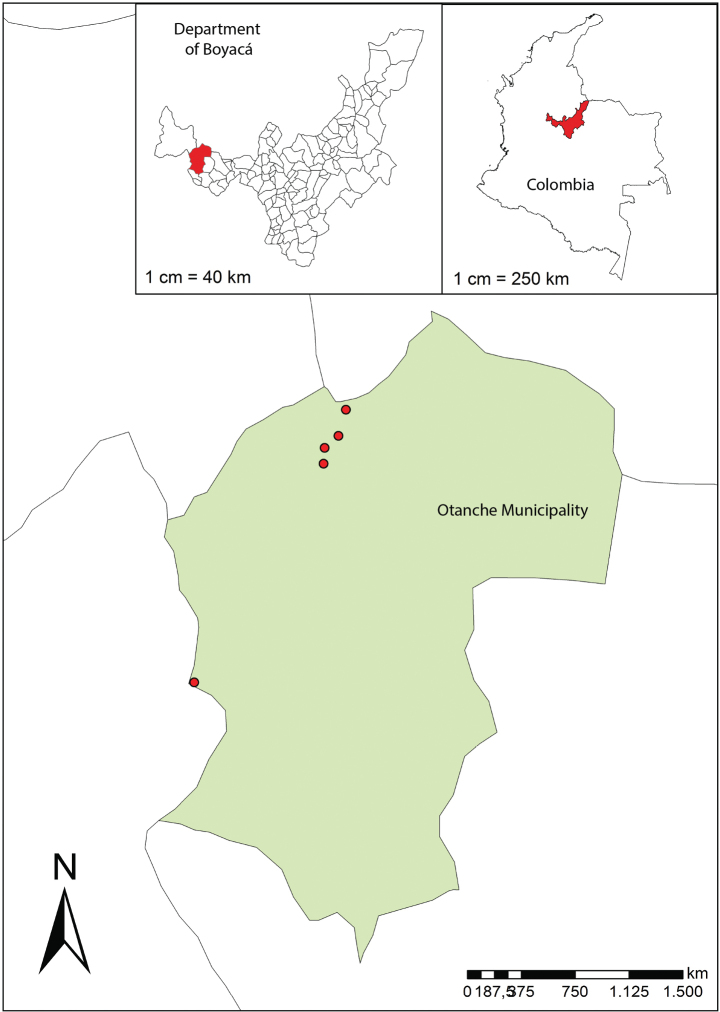
Geographic distribution map of *Piperquinchasense* M.A.Jaram.

Specimens were collected in Serranía de las Quinchas, located in the middle Magdalena Valley in Colombia, in the department of Boyacá. The sites visited range from 800–1200 m. a. s. l., the locality is dominated by humid montane forest (Fig. 1). Annual rainfall is 3333 mm on average; two dry seasons occur in January-February and June-August, and the average temperature is 26 °C ( Instituto de Hidrología, Meteorología y Estudios Ambientales, IDEAM). Detailed observations in the field, combined with examination of available herbarium collections, allowed the description of growth habits and phenological stages. Voucher specimens were deposited in the following herbaria HUA, H-UPTC, and UMNG-H (herbarium acronyms according to Index Herbariorum [Thiers continuously updated], and Instituto Humboldt – Red Nacional de Colecciones [http://rnc.humboldt.org.co/wp/]). In addition, taxonomic literature on *Piper* was examined (Trelease and Yuncker 1950, Steyermark 1984, Callejas-Posada 2020). The measurements included in the description below are based on herbarium specimens collected by the authors. Conservation status assessments employ the categories and criteria of the IUCN (2012, 2022). We calculated the extent of occurrence and area of occupancy using the R package *ConR* (Dauby et al. 2017).

For preparing the key of Neotropical *Piper* species with peltate leaves we used the literature (Trelease and Yuncker 1950, Steyermark 1984, Callejas-Posada 2020, Carvalho-Silva et al. 2022) and type specimens were examined using digitized plant specimens available on-line from JSTOR Global Plants (https://plants.jstor.org/).

We extracted DNA from silica gel dried tissue, using the DNAeasy plant mini kit (Qiagen, Valencia, California, USA). The ITS region was amplified using one of two pairs of primers ITS5-ITS4, or LEU1-ITS4 (Baldwin 1992). Sequencing was contracted with GenCore (Universidad de los Andes, Bogotá, Colombia). The resulting sequences (Table 1) were manually aligned against previously obtained alignments (Jaramillo et al. 2008). We selected 43 ITS sequences from the large alignment to portray here. Three sequences from *Piper* species from Asia and the South Pacific were used as outgroups. Forty sequences of Neotropical *Piper*, comprising representatives of all clades, and 14 species with peltate leaves were selected. Maximum Likelihood (ML) phylogenetic and bootstrap (100 replicates) analyses were conducted using RAxML (Stamatakis 2014).

**Table 1.** Genbank accessions for the new species of *Piper*.

Taxon	Voucher	Genbank Accession #
*Piperquinchasense* M. A. Jaram.	MAJ1807	OQ354973
	MAJ1939	OQ354974

## ﻿Taxonomic treatment

### ﻿ Piper quinchasense

Taxon classificationPlantaePiperalesPiperaceae

M.A.Jaram. sp. nov.

6EF27235-97B6-5E05-8161-C2FE5A667C65

urn:lsid:ipni.org:names:77320207-1

#### Type.

**Figure 2.**
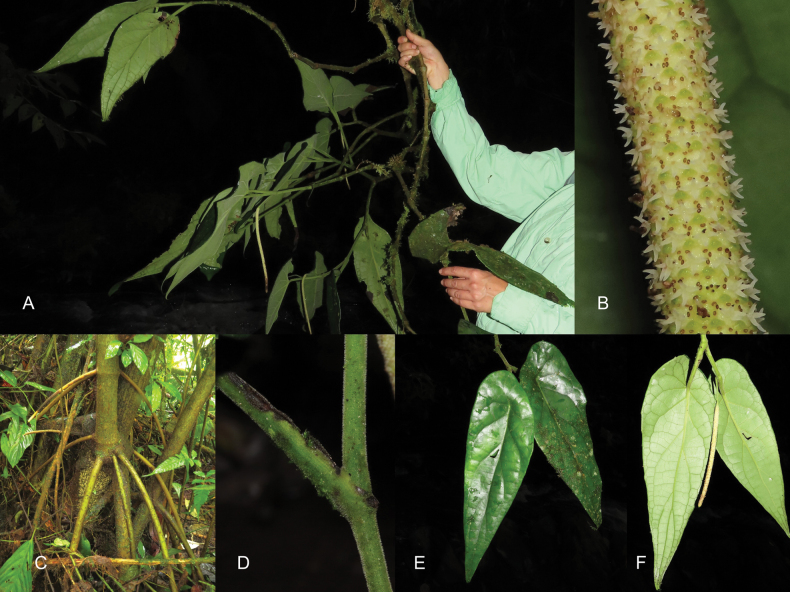
*Piperquinchasense* M. A. Jaram. **A** habit **B** magnified view of inflorescence **C** stilt roots **D** sheathing petiole **E** adaxial surface of leaves **F** abaxial surface of leaves and inflorescence. Photographs by D. Rodríguez-Duque.

**Figure 3.**
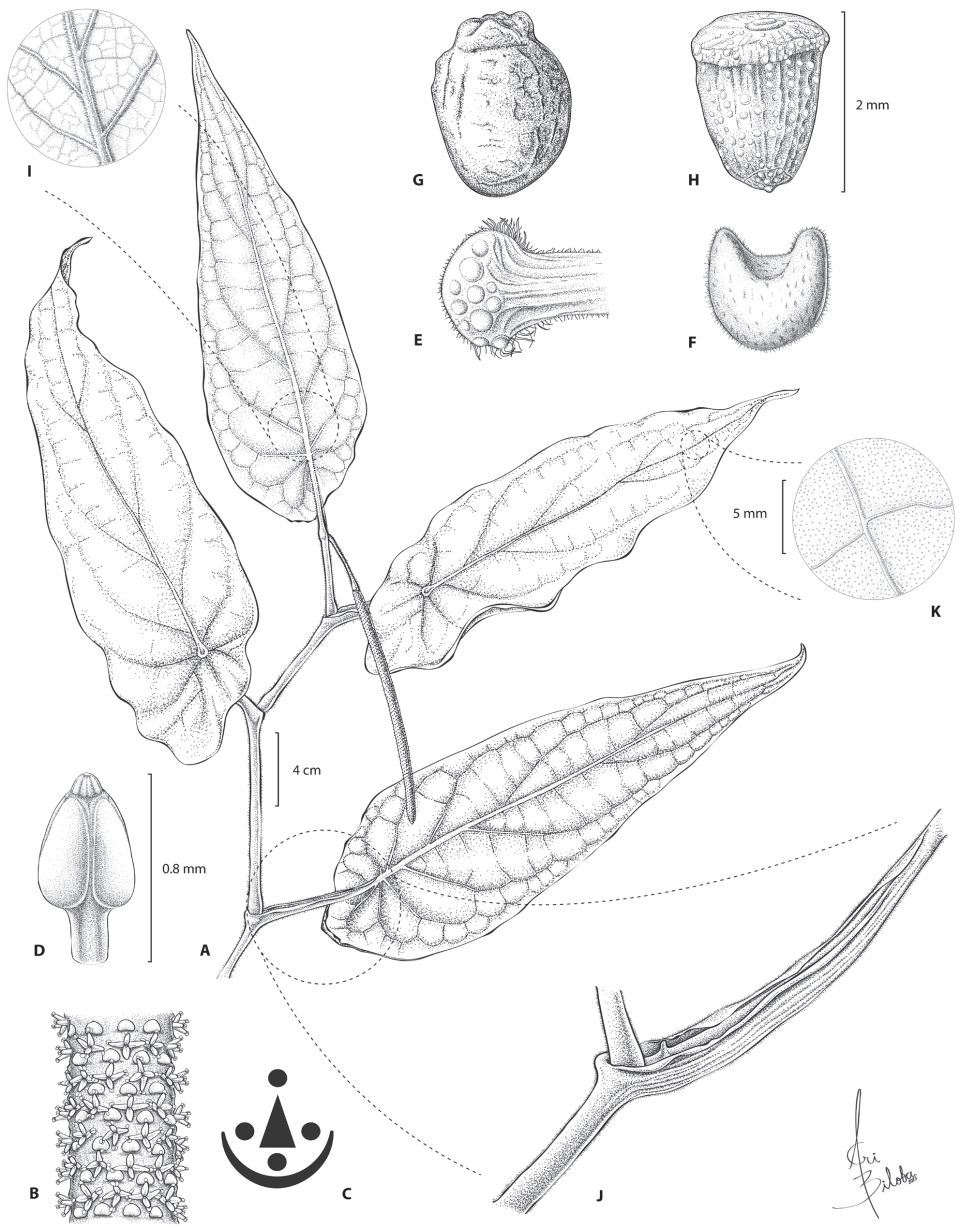
*Piperquinchasense* M. A. Jaram. **A** sympodial branch, showing both the abaxial and adaxial surface of the leaves **B** magnified view of inflorescence **C** floral diagram **D** anther **E** floral bract, abaxial view **F** floral bract view from above **G** fruit **H** seed **I** magnified view of leaf abaxial surface **J** sheathing petiole **K** magnified view of leaf adaxial surface. Illustration by Ariadna Valenzuela, based on M. Escobar-Alba 764, and photographs by D. Rodríguez-Duque.

Colombia. Boyacá: Otanche, vereda Las Quinchas, Sector La Y, Finca Lote Terreno, 5°48'17"N, 75°15′24"W, 1210 m, 17 Mayo 2022 [fl], M. A. Jaramillo et al. 1807 (holotype: HUA; isotypes: UPTC, UMNG-H). Figs 1–3.

#### Description.

*Piperquinchasense* is similar to *P.parianum*, it differs from the latter in having all leaf blades peltate (vs. leaves deeply lobed to peltate), and inflorescence peduncle 4–5 cm long, (vs. peduncle 1–2.7 cm long).

***Shrub***, 3 m tall, branched in the upper portion only, exhibiting stilt roots (Fig. 2C). ***Internodes*** 5–10 × 2.5–4.6 cm, green when young and becoming brown when maturing, tomentose, idioblasts not evident. ***Prophylls*** not seen. ***Petioles*** 4.5–8.5 cm long, vaginate the entire length (Figs 2D, 3J), tomentose, idioblasts evident. ***Leaf*** blades (28) 35–49 × 7.5–19 cm, oblong-lanceolate, base obliquely-peltate, asymmetric to truncate, peltate, petiole inserting 2.5–10.5 cm from the margin, on the adaxial surface leaf blade is depressed and umbonate above petiole insertion (Fig. 2E), blade medially asymmetric, apex long attenuate, green on the adaxial surface and green-silver on the abaxial surface when alive (Fig. 2E, F), coriaceous, chartaceous when dry, drying dark maroon on the adaxial surface and ochre on the abaxial surface, glabrous with visible idioblasts on the adaxial surface (Fig. 3K), sparsely tomentose on the blade and veins densely tomentose on the abaxial surface (Fig. 3I), eciliate, margin folds towards the abaxial surface appearing to form irregular spaced teeth; pinnately nerved to the distal third, 4–6 pairs of secondary veins, curved and ascending, diverging in angles that decrease towards the apex (from 80–30 degrees) and spacing slightly decreasing towards the base, tertiary veins forming areoles 1.2–1.8 × 0.5–1 cm, rectangular and not uniform in size, perpendicular to secondary nerves, nerves not impressed on the adaxial surface, elevated on the abaxial surface. ***Inflorescences*** a simple spike, terminal, pendulous; peduncle 4.5–5 cm long, tomentose, green, idioblasts not evident; rachis length in flower 12–19 cm × 3–4 mm, rachis length in fruit 25–27cm. ***Floral bracts*** cucullate, sagitate from above (Fig. 3F), 1.8–2.3 × 0.9–1.0 mm, pellucid dotted, pedicel fimbriate on the distal portion (Fig. 3E), forming bands around the spike (Fig. 3B). ***Flowers*** sessile with 4 stamens (Fig. 3C), filaments 0.25–0.40 mm long, anthers 0.3–0.6 × 0.16–0.26 long, with connective glabrate, longitudinally dehiscent, dithecous (Fig. 3D), ovary four carpellate, four stigmas sessile, not persistent in fruit, 0.4–0.5 mm long (Figs 2B, 3G). ***Fruits*** obpyriform, 2.1–2.4 × 1.0–1.6 mm, glabrous, green when alive, brown when dry (Fig. 3G). ***Seeds*** smooth, pellucid dotted, obpyriform, 1.9–2.1 × 1.0–1.3 mm, glabrous, brown when dry (Fig. 3H).

#### Phylogenetic relationships.

**Figure 4.**
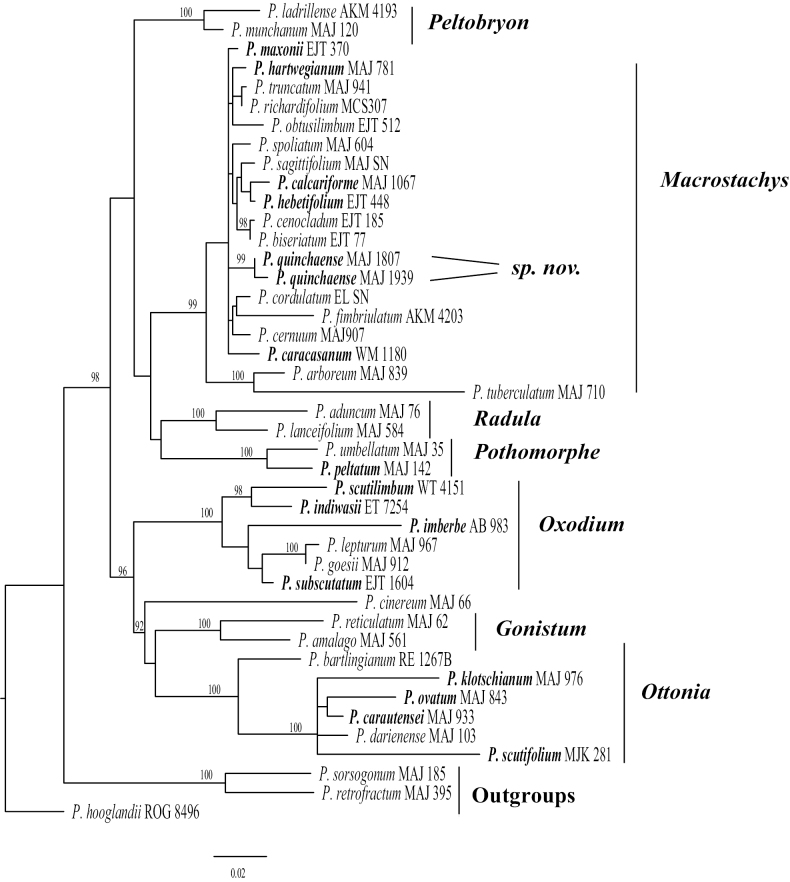
Phylogenetic relationships of *Piperquinchasense* M. A. Jaram. and 14 species of Neotropical *Piper* with peltate leaves (in bold). Topology based on Maximum likelihood analysis of nrITS sequences. Numbers on branches are maximum likelihood bootstrap support values (>90%).

*Piperquinchasense* belongs to the *Macrostachys* clade (Fig. 4). A group of shrubs and treelets reaching 7–8 (15) m tall, petioles sheathing above the middle or throughout their length, pinnately nerved leaves, mostly larger than 30cm long, inflorescences mostly pendulous (erect in some species), and flowers forming bands around the spike (Jaramillo et al. 2008). Species with peltate leaves have evolved independently in clades *Macrostachys*, *Pothomorphe*, *Oxodium* (=*Schilleria*, Callejas 2020) and *Ottonia*. Peltate leaves are known to occur in the two large genera of Piperaceae: *Piper* and *Peperomia*. They are more common in plants that grow in the shade of humid tropical forests (Wunnenberg et al. 2021). There is still much to learn about the functional morphology and anatomy of *Piper* species with peltate leaves.

#### Distribution and habitat.

The species is only known from the type locality Serranía de las Quinchas (Fig. 1). *Piperquinchasense* is a tall shrub in the understory of very humid forests; it often grows near streams. The occurrence of stilt roots (Fig. 2C), a character not commonly seen in *Piper*, suggests it is adapted for flooded areas near watercourses. It also is a very resilient plant that will produce adventitious roots if tumbled and it will continue growing, or even resprout, from a fallen leaf. Resprouting is common among shade-tolerant *Piper* (Lasso et al. 2009).

#### Phenology.

Flowering specimens were collected in March, and May. Fruiting specimens were collected in October.

#### Etymology.

The epithet *quinchasense*, refers to Serranía de las Quinchas, the type locality for this species. According to locals, Quinchas derives from the indigenous groups “Quinchos” that inhabited the region.

#### Conservation status.

This species is known only from one population in the type locality (Fig. 1). The extent of occurrence (EOO) of 8 km^2^ and area of occupancy (AOO) of 8 km^2^ are very small. The locality is under threat of disappearing for the extension of agricultural and mining activities (Rodríguez-Duque et al. 2021), which suggests it is Endangered [EN B1a]. Serranía de las Quinchas was declared a Regional Park in 2008; since its creation, the park extension has been reduced by 20% as the buffer zones were eliminated. Furthermore, the constant threat of coal mining makes the locality highly exposed to deforestation.

#### Additional specimens examined.

Colombia. —Boyacá: Otanche, vereda Las Quinchas, Sector La Y, Finca Lote Terreno, 5°48'17"N, 75°15′24"W, 26 October 2022, [fr] M. A. Jaramillo et al 1939 (HUA, UMNG-H); Boyacá: Otanche, Parque Regional Natural, Serranía de las Quinchas, 5°48′45.5"N, 75°15′22.2"W, [st] 14 June 2021, Magda Escobar-Alba *et al*. 489 (UPTC); Parque Regional Natural Serranía de las Quinchas, vereda las Quinchas, Finca Chorro Negro, 5°49′7.2"N, 75°14′57.3"W, [fl] 3 March 2022, Magda Escobar-Alba *et al*. 762 (UPTC).

#### Notes.

*Piperquinchasense* is a handsome species that differs from related Macrostachys taxa in having long lanceolate-oblong leaves. It is morphologically similar to *P.parianum* from which it differs in having mononomorphic leaves (all of them peltate) vs. leaves dimorphic, some peltate and others are deeply lobed. *P.parianum* is only known in the isolated cloud forests of “Peninsula de Paria”, a region located in the eastern portion of Coastal Cordillera in the extreme northeast of Venezuela. The flora of Paria Peninsula is characterized by the high occurrence of endemic species (Steyermark and Agostini 1966; Meier 2011) and several types of vegetation communities (Beard 1946). The flora of this region has many affinities to plants of Trinidad and Tobago (*Selaginellahartii* Hieron.; – Selaginellaceae; *5* Turrill – Acanthaceae: sensu Baksh-comeau et al. 2016), and the forests of the Northeastern sector of Guayana Shield in Venezuela and the Guianas (e.g., *Besleriainsolita* C.V. Morton – Gesneriaceae; Meier 2011). Serranía de las Quinchas, on the other hand, has the most floristic affinities with the Chocó region (e. g. *Dimerocostuscryptocalyx* N. R. Salinas & Betancur, Salinas and Betancur 2004). The regions have similar habitats driven by high precipitations despite their divergent biogeographical affinities.

A key to species of Neotropical *Piper* with peltate leaves is presented below.

##### ﻿New status

While preparing the key for peltate Neotropical *Piper*, we realized some species deserve new status. New status and new names are proposed for two species.

### ﻿ Piper neovenezuelense

Taxon classificationPlantaePiperalesPiperaceae

M.A.Jaram., stat. nov et nom. nov.

858148C6-E24B-52DB-B01E-FE1F9ED77E1A

urn:lsid:ipni.org:names:77320208-1

#### Basionym.

PiperveraguenseC. DC.var.venezuelense Steyerm. Fl. Venez. 2(2): 590 (1984). Type: Venezuela, Edo. Trujillo, 16 km de Boconó a lo largo de la carretera a Biscucuy, 1850 m, 11 aug 1964, F. Breteler 4082 (Holotype; VEN; Isotype: MER). Non *Pipervenezuelense* C.DC., J. Bot. 4: 216 (1866).

#### Note.

The epithet *P.neovenezuelense* is proposed here to replace PiperveraguenseC. DC.var.venezuelense Steyerm. Because *Pipervenezuelense* C. DC. (De Candolle 1866: 216) is already in use.

#### Etymology.

The new epithet *neovenezuelense* honors the intention of J. Steyermark to highlight the occurrence of this species in Venezuela.

Steyermark (1984) synonymized under *P.veraguense* C. DC. various distinct species: *P.albert-smithii* Trel. & Yunck., and *P.mutisii* Trel. & Yunck., and proposed that *P.veraguense* C. DC. has three varieties: P.veraguensevar.veraguense, P.veraguensevar.mutisii (Trel. & Yunck.) Steyermark, and he added P.veraguensevar.venezuelense Steyermark (Steyermark 1984). These species are similar in their overall morphology and potentially form a species complex. Unfortunately, we do not have sequence data to test the latter hypothesis. Here we consider them separate species. *P.veraguense* has glabrous leaves with nerves puberulent on the abaxial surface, not pellucid dotted (Callejas-Posada 2020); *P.albert-smithii* is distinct because it has fleshy warts below the nodes, and leaves are glabrous (Trelease and Yuncker 1950); and *P.mutisii* has fleshy warts below the nodes, nerves are dense velvety puberulent on abaxial surface; *P.neovenezuelense* has glabrous internodes without warty outgrowths, and nerves pilose on abaxial surface.

### ﻿ Piper andersii

Taxon classificationPlantaePiperalesPiperaceae

M.A.Jaram., stat. nov. et nom. nov.

E36F9ACE-BF8C-5D05-8239-545E32CA1C91

urn:lsid:ipni.org:names:77320209-1

Piper mikanianum Steud. var. peltatum Yunck., Bol. Inst. Bot. (São Paulo) no. 3: 54 (1966). Type: Brazil, Minas Gerais, Caldas, A. F. Regnell II 256*, 9 Jul 1864. Non Piperpeltatum L.

#### Note.

The epithet *P.andersii* is proposed here to replace PipermikanianumSteud.var.peltatum Yunck. because *Piperpeltatum* L., (Sp. Pl. 1: 30 1753) is already in use.

#### Etymology.

The new epithet *andersii* honors Anders Fredrik Regnell (1807—1884), Swedish physician and botanist who established himself in Minas Gerais (Brazil) and collected the type specimen for this species.

T. Yuncker provided a key to four varieties of *Pipermikanianum* (Kunth) Steud: P.mikanianum(Kunth)Steudvar.mikanianum; P.mikanianumf.clausum characterized by the closed sinus and overlapping lobes; P.mikanianumvar.pilosius C. DC. with leaves and stems strongly pilose with hairs up to 1mm long; and P.mikanianumvar.peltatum Yunck with peltate leaves and nerves minutely hirtellous on the abaxial surface (Yuncker 1972). Yuncker had mentioned that this latter variety might deserve species rank, we propose to make the change suggested and propose the new name *P.andersii*. Furthermore. *P.mikanianum* occurs in the states of Minas Gerais, Paraná and Rio Grande do Sul in Brazil and in Argentina, *P.andersii*, with peltate leaves has only been registered from the state of Minas Gerais.

### ﻿Key to Neotropical *Piper* species with peltate leaves

**Table:** 

1	Leaves subpeltate, petiole inserted slightly inside the leaf margin	**2**
–	Leaves peltate, petiole inserted 0.5–11 cm from the leaf margin	**12**
2	Leaves ovate-lanceolate or elliptic, up to 18 cm long, petioles terete	**3**
–	Leaves ovate, ovate –oblong or elliptic, more than 20 cm long, petioles sheathing	**5**
3	Inflorescence a raceme with glabrous to sparsely pubescent rachis	**4**
–	Inflorescence a spike, with pubescent rachis	***P.klotzschianum* (Kunth) C. DC.**
4	Leaves elliptic and glabrous without hirtellous intramarginal nerve	***P.brumadinense* M. Carv.-Silva & E. Guim.**
–	Leaves ovate, intramarginal nerve hirtellous on abaxial surface	***P.ovatum* Vahl**
5	Petioles sheathing only at the base, nerves puberulent on the abaxial surface	**6**
–	Petioles sheathing over half their length, leaves and nerves glabrous or pubescent	**7**
6	Leaves 35–20 cm long, base lobate	***P.omega* Trel.**
–	Leaves 10–20 cm long, base barely cordate	***P.marginecontinuum* Callejas**
7	Leaf base rounded or cordate, inflorescences erect	**8**
–	Leaf base lobate, inflorescences pendulous	**9**
8	Leaf ovate, leaf base rounded, peduncle up to 0.5 cm long	***P.palenquense* Callejas**
–	Leaf broadly ovate, leaf base deeply cordate, sinus open, peduncle 0.7–1 cm long	***P.vallicola* C. DC.**
9	Leaves more than 35 cm long, inflorescences 50–60 long	**10**
–	Leaves up to 26 cm long, inflorescences up to 20 cm long	**11**
10	Leaves up to 50cm long, sinus closed, longer lobe overlapping the petiole, peduncle 1.5–2.7cm long	***P.caracasanum* Bredem. ex Link**
–	Leaves up to 35cm long, sinus open, peduncle 7 cm long	***P.gualeanum* C. DC.**
11	Petioles shortly pubescent, inflorescences 10–20 cm long	***P.calcariforme* Tebbs**
–	Petioles with trichomes forming lines, inflorescences 6–9 cm long	***P.hebetifolium* W. C. Burger**
12	Plants herbaceous, inflorescences spikes, arranged in umbels	***P.peltatum* L.**
–	Shrubs, suffrutex, or climbers, inflorescences solitary racemes or spikes	**13**
13	Leaves 10–30 cm long, inflorescences erect	**14**
–	Leaves 35 cm long or longer, inflorescences erect or pendulous	**31**
14	Lianescent vines	**15**
–	Shrubs or suffrutex	**17**
15	Internodes, petioles and leaves glabrous, leaf blade ovate or oblong-elliptic, smooth	**16**
–	Internodes, petioles and abaxial leaf surface pilose, leaf blade obovate, slightly bullate	***P.parmatum* Dressler**
16	Leaf blades with evident idioblasts on abaxial surface, spikes 1–2 cm long, with obtuse apices	***P.foreroi* Gentry**
–	Leaf blades without visible idioblasts, spikes 5–6 cm long with mucronate apices	***P.peltifolium* Callejas**
17	Leaf base lobed or cordate	**18**
–	Leaf base rounded, obtuse or scutellate	**24**
18	Leaves rounded –ovate, leaf base deeply cordate	***P.andersii* M.A.Jaram., stat nov. nom nov.**
–	Leaves ovate, leaf base cordate or lobed	**19**
19	Shrubs densely crisp –villous	***P.copeyanum* (C.DC) Trel.**
–	Shrubs glabrous	**20**
20	Idioblasts visible on both surfaces	***P.subscutatum* (Miq.) C. DC.**
–	Idioblasts not visible	**21**
21	Petioles vaginate at the base, flowers not forming bands on the spikes	**22**
–	Petioles vaginate half the length or more, flowers forming bands on the spikes	***P.maxonii* C. DC.**
22	Leaves 5–10 cm wide, petiole inserted ca. 1 cm from the margin	***P.jacaleapaense* Callejas**
–	Leaves 15 –20 cm wide, petiole inserted 5–6 cm from the margin	**23**
23	Leaves broadly ovate, glabrous on both surfaces	***P.veraguense* C. DC.**
–	Leaves elliptic-oblong to ovate, nerves on the abaxial surface pilose	***P.neovenezuelense* M.A.Jaram., stat. nov. nom nov.**
24	Leaves glabrous	**25**
–	Leaves pubescent at least abaxially on veins	**27**
25	Leaves oblong-lanceolate, leaf base rounded	***P.imberbe* Trel.**
–	Leaves ovate, leaf base obtuse or spatulate	**26**
26	Leaf base narrowly spatulate	***P.indiwasii* W. Trujillo & M.A.Jaram.**
–	Leaf base broadly obtuse	***P.scutilimbum* C. DC.**
27	Apex short acuminate, inflorescence obtuse	**28**
–	Apex long attenuate, inflorescences mucronate	***P.tuerckheimii* C. DC.**
28	Flowers forming bands around the spike	***P.hammelii* Callejas**
–	Flowers laxly arranged in a spike or raceme	**29**
29	Plant glabrous, inflorescences a spike	***P.scutifolium* Yunck.**
–	Villous shrub, inflorescence a raceme	**30**
30	Petiole inserted ca. 1 cm from margin, with visible idioblasts on leaf lamina	***P.cariacicaense* M. Carv.-Silva & E.F.Guim.**
–	Petiole inserted 0.5 cm from margin, without visible idioblasts on leaf lamina	***P.carautensei* E.F.Guim. & M. Carv.-Silva**
31	Leaves broadly ovate, petiole vaginate to the middle	**32**
–	Leaves ovate, lanceolate or oblong, petiole vaginate above the middle	**33**
32	Leaf base rounded, inflorescence short-apiculate	***P.mutisii* Trel. & Yunck.**
–	Leaf base cordate, inflorescence obtuse	***P.grandilimbum* C. DC.**
33	Internodes warty above nodes, leaves glabrous	***P.albert-smithii* Trel. & Yunck.**
–	Internodes smooth, leaves pubescent or tomentose, at least on the abaxial surface	**34**
34	Leaf strongly bullate	***P.hartwegianum* (Benth.) C. DC.**
–	Leaves not bullate, or occasionally softly bullate	**35**
35	Leaves elliptic-ovate, apex acute or short acuminate	**36**
–	Leaves oblong-lanceolate, apex gradually acuminate or long attenuate	**37**
36	Heteromorphic trichomes of intermixed on the abaxial surface, inflorescence up to 43 cm long	***P.peltilimbum* Yunck.**
–	Trichomes of uniform length on the abaxial surface, inflorescences 50–60 cm long	***P.candollei* Sodiro**
37	Leaves 14–26 cm wide, leaf base deeply cordate sub-peltate to obliquely peltate	***P.parianum* Yunck.**
–	Leaves 7.5–19 cm wide, leaf base obliquely peltate	***P.quinchasense* M.A.Jaram., sp. nov.**

## Supplementary Material

XML Treatment for Piper quinchasense

XML Treatment for Piper neovenezuelense

XML Treatment for Piper andersii

## ﻿Additional information

### Author ORCIDs

M. Alejandra Jaramillo https://orcid.org/0000-0002-6539-4149

Dayro Rodríguez-Duque https://orcid.org/0000-0002-3829-3377

Magda Escobar-Alba https://orcid.org/0000-0002-3756-9132

### Data availability

All of the data that support the findings of this study are available in the main text or Supplementary Information.
